# Curcumenol Mitigates the Inflammation and Ameliorates the Catabolism Status of the Intervertebral Discs *In Vivo and In Vitro via* Inhibiting the TNFα/NFκB Pathway

**DOI:** 10.3389/fphar.2022.905966

**Published:** 2022-06-20

**Authors:** Xiao Yang, Baixing Li, Haijun Tian, Xiaofei Cheng, Tangjun Zhou, Jie Zhao

**Affiliations:** Shanghai Key Laboratory of Orthopedic Implants, Department of Orthopedics, Ninth People’s Hospital, Shanghai Jiaotong University School of Medicine, Shanghai, China

**Keywords:** curcumenol, intervertebral disc degeneration, nucleus pulposus, TNFα/NFκB pathway, lumbar spine instability mouse model

## Abstract

Low back pain (LBP) caused by intervertebral disc degeneration (IVDD) is accredited to the release of inflammatory cytokines followed by biomechanical and structural deterioration. In our study, we used a plant-derived medicine, curcumenol, to treat IVDD. A cell viability test was carried out to evaluate the possibility of using curcumenol. RNA-seq was used to determine relative pathways involved with curcumenol addition. Using TNFα as a trigger of inflammation, the activation of the NF-κB signaling pathway and expression of the MMP family were determined by qPCR and western blotting. Nucleus pulposus (NP) cells and the rats’ primary NP cells were cultured. The catabolism status was evaluated by an *ex vivo* model. A lumbar instability mouse model was carried out to show the effects of curcumenol *in vivo*. In general, RNA-seq revealed that multiple signaling pathways changed with curcumenol addition, especially the TNFα/NF-κB pathway. So, the NP cells and primary NP cells were induced to suffer inflammation with the activated TNFα/NF-κB signaling pathway and increased expression of the MMP family, such as MMP3, MMP9, and MMP13, which would be mitigated by curcumenol. Owing to the protective effects of curcumenol, the height loss and osteophyte formation of the disc could be prevented in the lumbar instability mouse model *in vivo*.

## Background

Over 632 million people were affected by low back pain (LBP) resulting from intervertebral disc degeneration (IVDD) across the world, in a global health report published in the year 2010, which is the leading reason contributing to disability (Chou et al., 2011; DePalma et al., 2011; Takatalo et al., 2011; Vos et al., 2012). In some developing countries like China, LBP ranks as the second leading cause of years lived with disability burden disease (Wu et al., 2019). Thus, the treatment of IVDD should be focused highly to try and solve this huge burden. The intervertebral disc (IVD) is a flexible joint between the vertebral bodies, that functions as a connective motif. They can afford axial compressive forces on the spine and transmit the load effectively, therefore accomplishing multi-axial flexibility of the spine (Pattappa et al., 2012; Sakai and Grad, 2015). IVD is composed of three parts: the central nucleus pulposus (NP), the surrounding annulus fibrosus (AF) and the cartilage endplate (CEP) covering the vertebral bodies. Degeneration of the discs is always initiated at the part of the central NP, displayed as tissue dehydration and shrinking, which is a progressive cell mediated cascade process. For example, some inflammatory pathways were activated during the degeneration and one of the most well-known signaling pathways is NF-κB, which is accompanied by the disturbance of metabolic homeostasis and the consequent extracellular matrix (ECM) degradation (Tang et al., 2018; Hu et al., 2020). Conventional strategies for IVDD include pharmacological treatment and invasive surgeries (Frapin et al., 2020). However, the former only focuses on symptomatic relief without structural reconstruction, and finally, fusion surgery is inevitable. Regrettably, there is still a lack of transitional treatment between the conservative management and the final surgery. Thus, from the prospective of molecular mechanisms affecting IVDD, inhibiting the inflammatory pathways like NF-κB may offer an alleviation on the progression of degeneration.

Curcumenol is a bioactive compound isolated from the edible rhizome of Curcuma zedoaria (zedoary, Zingiberaceae) (Hikino et al., 1968; Xu et al., 2015). A variety of herbs, which are widely used as traditional treatments for inflammatory pain in ancient society, possess this important constituent (Assis et al., 2013; Saikia et al., 2015). Recent studies have purified this compound and confirmed that it shows various functions like anti-inflammatory, neuroprotective, and antioxidant activities (Sun et al., 2010; Pintatum et al., 2020; Yang et al., 2021). Thus, in our study we consider further expanding the application of curcumenol in IVDD.

In our research, we explored the possibility of curcumenol to treat inflammation of IVD, especially in lumbar instability induced degeneration *in vivo* and in inflammatory model of the rat’s *ex vivo* IVD culture. Moreover, we showed effective inhibition on the activation of TNFα/NF-κB pathway in NP cell lines and primary NP cell *in vitro*.

## Methods

### Isolation and Cell Culture of Rats’ Primary Nucleus Pulposus Cells

6-week-old male Sprague-Dawley rats (Shanghai Lab, Animal Research Center Co., Ltd., Shanghai, China) were killed by cervical dislocation and put in 75% ethanol for 10 min (Sengupta, 2013). Their tails were dissected with skin flayed, then the intervertebral discs were cut away from the CEP of the disc, then the NP cells were extracted to be soaked in the 1% collagenase II solution for 2 h, followed by centrifugation (in 300 x g, 37°C for 5 min) and suspension, the primary NP cells were cultured in Dulbecco’s Modified Eagle’s Medium (DMEM) supplemented with 10% FBS and 1% penicillin-streptomycin (Gibco, Thermo Fisher Scientific, Waltham, MA, United States) at 37°C with 5% CO_2_.

### Culture of Nucleus Pulposus Cell Lines

The rat’s NP cells are immortalized cell lines (Oh et al., 2016), which were kindly gifted by Dr. Chen Di at the Department of Orthopedic Surgery, Rush University Medical Center (Chicago, IL, United States). Cells were maintained in Dulbecco’s modified Eagle’s medium (DMEM) supplemented with 10% FBS and 1% penicillin-streptomycin (Gibco, Thermo Fisher Scientific, Waltham, MA, United States) at 37°C with 5% CO_2_.

### RNA Extraction and Real-Time Quantitative PCR Analyses

NP cell line and primary NP cells were stimulated with TNFα (10 ng/ml) with different concentrations of curcumenol (0, 6.25, 12.5, 25, and 50 μM, dissolved in DMSO; bought from Selleck Chemicals, Houston, TX, United States; with the following characteristics: high performance liquid chromatography, purity = 99.89%; nuclear magnetic resonance, consistent structure) for 24 h at 37°C with 5% CO_2_. Then, total RNA was isolated from the cells using TRIzol reagent (Thermo Fisher Scientific, Waltham, MA, United States) as per the manufacturer’s protocol. First strand complementary DNAs (cDNAs) were reverse transcribed from the extracted RNAs using the cDNA Synthesis Kit (Takara Bio, Otsu, Japan). Real-time qPCR was conducted using the TB Green Premix Ex Taq Kit (Takara Bio) on an Applied Biosystems QuantStudio 6 Flex Real-Time PCR System (Thermo Fisher Scientific) per the following conditions: denaturation at 95°C for 30 s; 40 cycles of 95°C for 3 s and 60°C for 34 s; and then 95°C for 15 s, 60°C for 60 s, and finally, 95°C for 15 s. Specific primer pairs were designed using NCBI BLAST and sequences provided in Table 1. The gene expression of β-actin was used as an internal control. Target gene expression levels were determined using the 2^−ΔΔCT^ method.

**TABLE 1:** Primers Information.

Gene	Accession number	Description	5′-primer-3′
Col2a1	NM_053304.1	F R	GGA​TCG​ACC​CTA​ACC​AAG​GC GAT​CGG​AAC​CTT​CGC​TTC​CA
MMP3	NM_133523.3	F R	TTT​GGC​CGT​CTC​TTC​CAT​CC GCA​TCG​ATC​TTC​TGG​ACG​GT
MMP9	NM_031055.2	F R	TCT​GCC​TGC​ACC​ACT​AAA​GG CAG​GCT​GTA​CCC​TTG​GTC​TG
MMP13	NM_133530.1	F R	TGC​TGC​ATA​CGA​GCA​TCC​AT TGT​CCT​CAA​AGT​GAA​CCG​CA
β-actin	NM_031144.3	F R	GTC​CAC​CCG​CGA​GTA​CAA​C GGA​TGC​CTC​TCT​TGC​TCT​GG
TRAF1	NM_001271240.2	F R	AGT​GCA​GCC​ACT​GAT​GGA​AT AGT​GCA​GCC​ACT​GAT​GGA​AT
TRAF 2	NM_001107815.2	F R	CGA​AGA​CCG​TTG​GGG​CTT​T TCG​TGG​CAG​CTC​TCG​TAT​TC
TRAF3	NM_001395112.1	F R	CCC​TCA​CTT​CTG​AGC​TTC​CC CTT​GGC​AGG​CTG​TGC​ATT​TT
TRAF4	NM_001107017.2	F R	CTA​CAA​GTT​CCT​GGA​GAA​GCC​C CCT​GTA​GGT​GAC​GAA​GTG​GC
TRAF6	NM_001107754.2	F R	GGG​GAG​CTT​TCT​AGT​CGG​TTG​T GGA​CAC​TTT​ACC​GTC​AGG​GAA
CXCL1	NM_030845.2	F R	ACA​CTC​CAA​CAG​AGC​ACC​AT TCG​CGA​CCA​TTC​TTG​AGT​GT
CXCL6	NM_022214.2	F R	TCA​AGC​TGC​TCC​TTT​CTC​GG AAC​GGA​GCT​TCT​GGG​TCA​AG
CXCL10	NM_139089.2	F R	ATG​ACG​GCT​CTC​CTA​GCT​CT CCT​TGG​GAA​GGT​GGT​GGT​AA
CXCL16	NM_001017478.1	F R	CAG​GAG​TCT​CGA​GTC​GGA​AG AGC​CGA​CAT​CCA​GCA​GAT​TC
NOS2	NM_012611.3	F R	TAG​TCA​ACT​ACA​AGC​CCC​ACG TTG​ATC​CTC​ACG​TGC​TGT​GG
IL1RL1	NM_013037.1	F R	AAC​TGG​TGT​GAC​CGA​CAA​GG AAT​CCC​CTG​GGA​CCA​AGC​TA

### RNA Seq

NP cell lines were stimulated with curcumenol (0 as control group and 50 μM as treatment group) for 24 h at 37°C with 5% CO_2_. Then, total RNA were isolated from the cells using TRIzol reagent (Thermo Fisher Scientific, Waltham, MA, United States) as per the manufacturer’s protocol and analyzed *via* RNA (transcriptome) sequencing by Wuhan Huada Gene Technology Co., Ltd. (China): using the Kyoto Encyclopedia of Genes and Genomes (KEGG) pathways, volcano plot (|log2FC| >=1, FDR <=0.001), and heat map by mRNA relative expression as transcripts per kilobase million (TPM) to further review the pathways involved in the Mybgi platform (WuhanHuada Gene Technology, https://mybgi.bgi.com/tech/login).

### Cell Viability Analysis

Cell viability following curcumenol treatment was evaluated using the Cell Counting Kit-8 (CCK-8; Dojindo Laboratories Co., Ltd., Kumamoto, Japan). NP Cell line and primary NP cells were seeded onto a 96-well plate at a density of 2 × 10^3^ cells/well the day before they were treated with increasing concentrations of curcumenol (0, 12.5, 25, and 50 μM) for 24, 48, and 72 h. NP and primary NP cells were cultured in DMEM supplemented with 10% FBS and 1% penicillin/streptomycin (complete DMEM). Cell media containing curcumenol and 1:1,000 DMSO were changed every 2 days. At the end of the experimental periods, cells were incubated with fresh complete media containing 10 μl of CCK-8 reagent for 1.5 h at 37°C. Complete media containing CCK-8 reagent but no cells and untreated cells were used as blank and mock controls, respectively. The absorbances (measured as optical density; OD) at 450 nm were measured on an Infinite M200 Pro multimode microplate reader (Tecan Life Sciences, Männedorf, Switzerland).

### Western Blot Analysis

To detect the expression of the MMP family on a protein level, NP cell line and primary NP cells were stimulated by TNFα (10 ng/ml) with or without curcumenol (50 μM) for 24 h at 37°C with 5% CO_2_. For preventive analysis of the NF-κB pathways, cells were pretreated with curcumenol (0 and 50 μM) for 2 h at 37°C, and then stimulated with TNFα for 10 min at 37°C; then, total cellular proteins were extracted from cultured cells using RIPA lysis buffer, supplemented with phosphatase and protease inhibitors (Roche, Basel, Switzerland). The protein was quantified by the BCA assay (Thermo Fisher Scientific, Inc.) and equal quantities of extracted proteins (20–30 μg) were resolved on a 10% or 12.5% SDS-PAGE gel and separated proteins were electroblotted onto 0.22 μM PVDF membranes (Merck-Millipore). Membranes were blocked with 5% BSA-PBS at room temperature for 1 h and then incubated with primary antibodies (diluted 1:1000 in 5% BSA-PBS) overnight (at least 16 h) at 4°C. Primary antibodies against p65 (D14E12; rabbit mAb), phospho-p65 (Ser536, 93H1; rabbit mAb), IκBα (L35A5; mouse mAb), phosphor- IκBα (Ser32; rabbit mAb), and β-actin (D6A8; rabbit mAb) were purchased from Cell Signaling Technology (Danvers, MA, United States). Primary antibodies against TRAF3 (ab239357; rabbit mAb), CXCL10 (ab9807; rabbit mAb), Col2a1 (ab188570; rabbit mAb), MMP3 (ab52915; rabbit mAb), MMP9 (ab58803; mouse mAb) and MMP13 (ab51072; rabbit mAb) were obtained from Abcam (Cambridge, United Kingdom). The membranes were then washed extensively in Tris-buffered saline-Tween20 (TBST) and subsequently incubated with anti-rabbit IgG (H+L) (DyLight™ 800 4× PEG Conjugate; Cell Signaling Technology) secondary antibody (1:5000 dilution) for 1 h at room temperature in the dark. The membranes were again extensively washed in TBST, and protein immunoreactivity were detected on a LI-COR Odyssey Fluorescence Imaging System (LI-COR Biosciences, Lincoln, NE, United States). Semi-quantitative analysis of protein immunoreactive band intensity was measured using ImageJ V1.8.0 software (National Institutes of Health) and normalized to the internal loading control β-actin.

### Intervertebral Disc *Ex Vivo* Model culture

All animal experiments were approved by the Institutional Animal Care and Ethics Committee of Ninth People’s Hospital, Shanghai Jiaotong University School of Medicine (Shanghai, China). A total of 18 male Sprague-Dawley rats (3 months old) were euthanized using carbon dioxide, and then their spinal columns were harvested under aseptic conditions (Dutta and Sengupta, 2016). The coccygeal discs (Co6/7, Co7/8) were harvested with soft tissues removed and intact endplates, then the discs were flushed three times using PBS containing 1% penicillin/streptomycin (Sigma-Aldrich, St. Louis, MO, United States). The discs with vertebral endplates were cultured in DMEM medium supplemented with 10% FBS and 1% penicillin-streptomycin (Gibco, Thermo Fisher Scientific, Waltham, MA, United States) at 37 C with 5% CO_2_. The medium was replaced every 3 days, and after 14 days’ of culture, the models were harvested and fixed in 4% PFA for subsequent studies.

### Animals and Surgical Procedures

The lumbar spine instability model, which had stable effects to induce IVDD in mouse, was used in these studies (Oichi et al., 2018; Liu et al., 2021). All animal experiments were approved by the Institutional Animal Care and Ethics Committee of Ninth People’s Hospital, Shanghai Jiaotong University School of Medicine (Shanghai, China) and performed in accordance with the principles and procedures of the National Institutes of Health (NIH) Guide for the Care and Use of Laboratory Animals and the guidelines for animal treatment of Shanghai Jiaotong University. 18 male 8-week-old C57/BL mice (Shanghai Lab, Animal Research Center Co., Ltd., Shanghai, China) were housed under pathogen-free conditions at 26–28°C and 50–65% humidity with a 12-h day/night cycle. The animals were fed standard rodent chow and had access to fresh water ad libitum. Before surgical procedures, mice were anesthetized by intraperitoneal injections of pentobarbital sodium (5 mg/100 g of body weight) and the fur on the skin was shaved, then a 3 cm-incision was made on the dorsal part and the spinous process was dissected in 12 of them with the others intact as sham groups. After the operation, the incisions were sutured, and the mice were cultured for another month with intraperitoneal injection of curcumenol [sham group: corn oil (cat. no. C8267; Sigma-Aldrich; Merck KGaA; 1 ml DMSO diluted in 100 ml corn oil); surgery group: corn oil; curcumenol group: 50 mg curcumenol pre-dissolved in 1 ml DMSO and then diluted in 100 ml corn oil]; two times a week at 4 mg/kg/time (Wang et al., 2021; Zhang et al., 2022). At the end of the experimental period, all mice were sacrificed, and the spine was extracted, cleaned of soft tissues, and the vertebral column fixed in 4% PFA.

### Histology and Immunofluorescence Staining

Fixed IVD tissue samples were embedded into paraffin blocks and then subjected to histological sectioning (8 μM thickness). For histological assessment, paraffin tissue sections were processed for Safranin O-Fast Green and hematoxylin and eosin (H&E) staining (Servicebio, Wuhan, China) at RT for 2–5 min, in accordance with the manufacturer’s instructions. The histological score was based on the modified histologic grading system (Ji et al., 2018). For tissue staining, the sections were de-paraffinized in graded xylene, rehydrated in graded alcohol solutions, and then incubated in antigen retrieval buffer (Roche Diagnostics) at 37°C for 30 min. After cooling to RT, the sections were immersed in PBS (pH 7.4) and washed three times for 5 min each. Then, auto-fluorescence quencher was added to the sections for 5 min, and then blocked with blocking buffer for 30 min at room temperature. Sections were subsequently incubated with primary antibodies in a wet box at 4°C overnight. Primary antibodies were used at 1:100 dilutions, including Anti-TNFα (cat. no. ab183218; Abcam), anti-IL-1β (cat. no. ab234437; Abcam), and anti-Col2a1 (cat. no. AF0135; Affinity). The next day, the sections were washed with PBS and then incubated with Alexa Fluor 594-conjugated secondary antibody (anti-rabbit, 1:500; Cell Signaling Technology) for 50 min at room temperature in the dark. The sections were washed with PBS and then incubated with DAPI solution (Sigma-Aldrich, St Louis, MO, United States) for 10 min in the dark to stain cell nuclei. Sections were subjected to final PBS washes, air-dried, and then sealed with anti-fluorescence quenching tablets. Digital fluorescence images were captured under a Leica DM4000 B epifluorescence microscope (Leica Microsystems) and IOD measurements were carried out using Image Pro Plus 6.0 software (Media Cybernetics, Inc.).

For immunofluorescence assessment of p-p65 translocation, NP cells were cultured on slides added to a 6-well plate. At 10% confluence, the cells were stimulated with TNFα for 20 min at 37°C, with or without curcumenol pretreatment for 2 h at 37°C. Then these cell slides were fixed with 4% PFA at RT for 2 h and then processed as slides as aforementioned.

### Immunohistochemistry

Fixed IVD tissue samples were embedded in paraffin and cut into slices (8 μM), then subjected to an immunohistochemistry kit (cat. no. G1215-200T; Wuhan Servicebio Technology Co., Ltd.) as per the manufacturer’s instruction. The primary antibodies include rabbit anti-TNFα (cat. no. ab9579; Abcam), anti-IL-1β (cat. no. ab283818; Abcam), and anti-Col2a1 (cat. no. ab34712; Abcam). Digital images were captured under a Leica DM4000 B microscope at ×10 and ×20 magnification, and positively stained cell measurements were obtained using Image Pro Plus 6.0 software.

### Radiographic Analysis

Digital X-ray imaging of the lumbar spine was conducted in the anteroposterior axis with a 21 lp/mm detector that provides up to ×5 geometric magnification (Faxitron VersaVision; Faxitron Bioptics LLC, Tucson, AZ, United States). The disc height index (DHI) was measured according to the formula provided previously (Che et al., 2020).

### Statistical Analysis

Three independent experiments or repeated measurements were conducted for all the data. Data are presented as the mean ± standard deviation (S.D.). Significant differences between study groups were obtained by one-way analysis of variance (ANOVA) with Tukey’s post hoc test. Significant differences in ordinal data between study groups were assessed by the Kruskal–Wallis test with a Dunn’s post hoc test. Analyses were conducted using SPSS 19.0 software (IBM Corporation, Armonk, NY, United States). Statistical significance was set if the *p* value < 0.05 unless otherwise indicated.

## Results

### Curcumenol Showed Little Cytotoxicity in Nucleus Pulposus Cells and Down-Regulated Inflammatory Pathways Based on RNA Seq *In Vitro*

**FIGURE 1:**
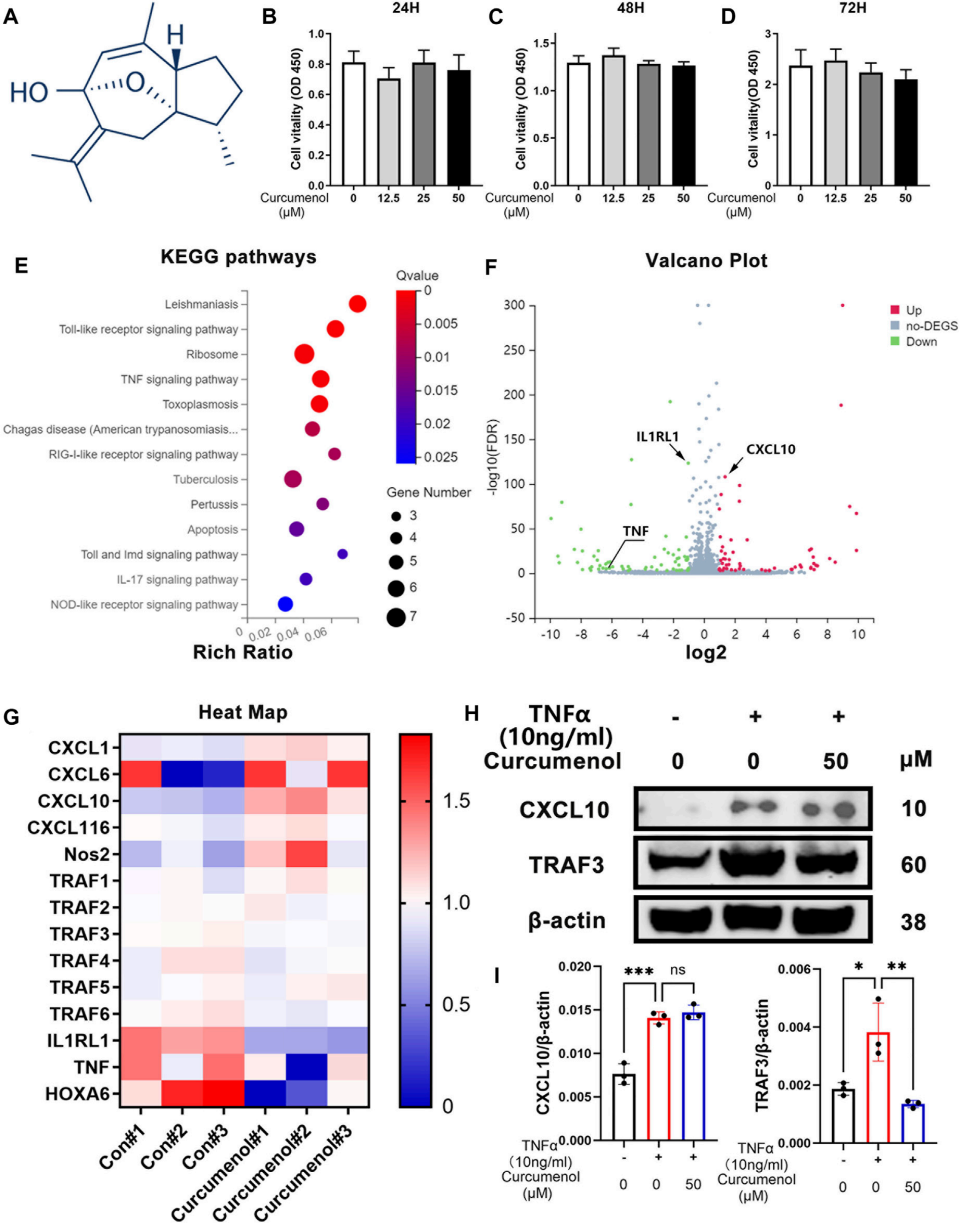
Curcumenol showed little cytotoxicity in NP cells and down-regulated inflammatory pathways based on RNA-seq *in vitro*. **(A)** Chemical structure of Curcumenol. **(B–D)** Cell Counting Kit-8 assay results of NP cells stimulated with Curcumenol at different concentrations (0, 12.5, 25, and 50 μM) and different time periods (ranging from 24 to 72 h). **(E)** Ratio of up-regulated mRNA in NP cells treated with Curcumenol (50 μM) versus DMSO (1:1000) using KEGG pathway analyses (3 paired biological replicates). **(F)** Ratio of changed mRNA in NP cells treated with Curcumenol (50 μM) versus DMSO (1:1000) using Volcano Plot analyses (3 paired biological replicates). **(G)** Heat Map of changed mRNA in NP cells treated with Curcumenol (50 μM) versus DMSO (1:1000) using Volcano Plot analyses (3 paired biological replicates). **(H)** Western blot analysis of CXCL10 and TRAF3 expression in NP cells stimulated with TNFα (10 ng/ml) or/and 50 μM Curcumenol for 24 h **(I)** RT-qPCR analysis of the relative mRNA expression levels of CXCL10 and TRAF3 expression in NP cells stimulated with TNFα (10 ng/ml) or/and 50 μM Curcumenol for 24 h. All data are presented as mean ± SD from three replicates. *p < 0.05, **p < 0.01, ***p < 0.001 and ****p < 0.0001.

The chemical structure of curcumenol is shown in Figure 1A. For security when using curcumenol to treat IVDD, at first, we used CCK-8 test to study if there was any cytotoxicity in NP cell line. With a concentration of 0, 12.5, 25, and 50 μM, curcumenol showed little cytotoxicity in NP cell line and did not affect the proliferation rate of these cells, ranging from 24 to 72 h (Figures 1B–D). So the NP cell line was treated with 0 and 50 μM curcumenol for 24 h and then went for subsequent RNA-seq. KEGG pathway analysis showed that the genes involved in inflammatory pathways changed significantly, including Toll-like receptor signaling pathway, IL-17 signaling pathway, and especially the TNF signaling pathway (Figure 1E). Moreover, curcumenol down-regulated TNF and IL1RL1 but up-regulated CXCL10 based on the volcano plot analysis (Figure 1F). Using heat map analysis to further explore the TNF signaling pathway and CXCL chemokine family, we found that CXCL1, 6, 10, 16, and Nos2 increased with curcumenol treatment, and TRAF1, 2, 3, 4, 5, 6, IL1RL1, TNF, and HOXA6 decreased (Figure 1G). To confirm this consequence, we used TNFα (10 ng/ml) to treat NP cell line with or without curcumenol (50 μM), and PCR test combined with western blot analysis both showed that TRAF3 increased with TNFα but could be effectively inhibited by curcumenol. On the contrary, CXCL10 increased with TNFα but did not decrease with curcumenol (Figures 1H,I). Further, we detected the other genes and found that the up-regulation of CXCL1, TRAF4, and NOS2 induced by TNFα could be inhibited by curcumenol significantly and CXCL6, CXCL16, TRAF1, TRAF2, and TRAF6 were mitigated, although without significance (Supplementary Figures S1A–C). By the way, curcumenol treatment could inhibit the expression of IL1RL1 significantly (Supplemenatry Figure S1D), meaning curcumenol played an important role in multiple inflammatory pathways, especially the TNFα signaling pathway.

### Curcumenol Inhibited the Phosphorylation of the NFκB Pathway and Up-Regulation of the MMP Family *In Vitro*

**FIGURE 2:**
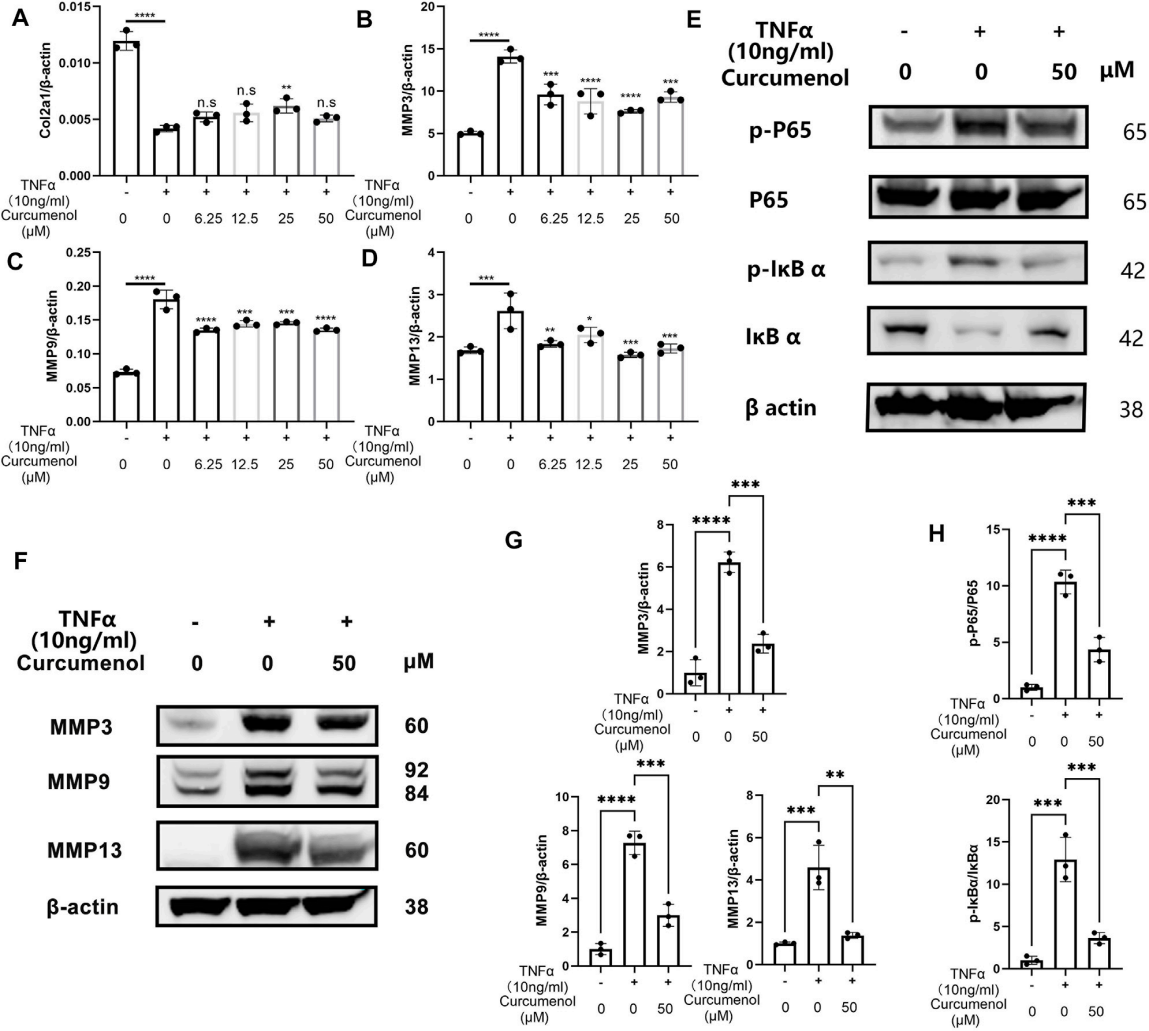
Curcumenol inhibited the phosphorylation of the NFκB pathway and up-regulation of the MMP family *in vitro*. **(A–D)** RT-qPCR analysis of the relative mRNA expression levels of Col2a1, MMP3, MMP9, and MMP13 in NP cells stimulated with TNFα (10 ng/ml) and different range of Curcumenol (0, 6.25, 12.5, 25, and 50 μM) for 24 h. **(E)** Western blot analysis of p-P65, P65, p-IκBα, and IκBα expression in NP cells pretreated with 50 μM Curcumenol and then stimulated with TNFα (10 ng/ml) for 10 min. **(F)** Western blot analysis of MMP3, MMP9, and MMP13 expression in NP cell stimulated with TNFα (10 ng/ml) or/and 50 μM Curcumenol for 24 h. **(G)** Semi-quantification of grey scale value in MMP3, MMP9, and MMP13 was conducted using β-actin as the reference in panel F. **(H)** Semi-quantification of grey scale value in p-P65/P65 and p-IκBα/IκBαwas conducted in panel E. All data are presented as mean ± SD from three replicates. *p < 0.05, **p < 0.01, ***p < 0.001, and ****p < 0.0001. p-, phosphorylated; Col2a1, collagen type II α 1 chain.

**FIGURE 3:**
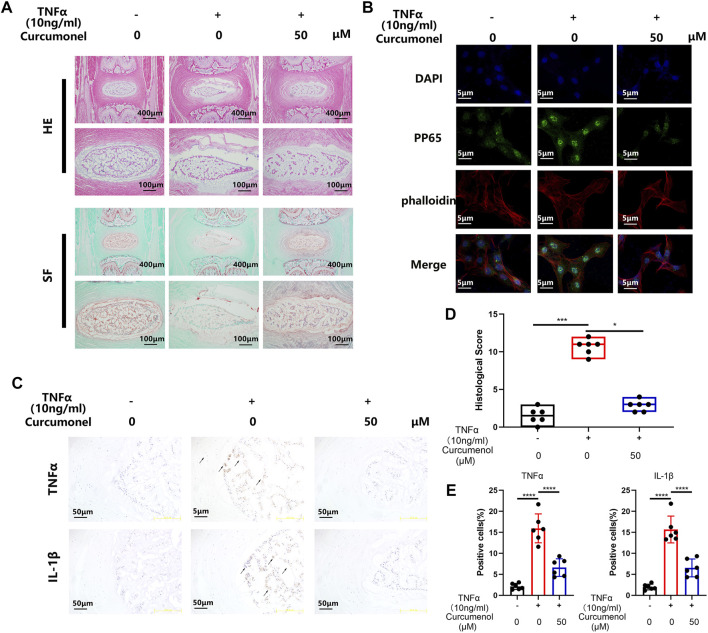
Curcumenol mitigated the inflammation induced by TNFα in Rat’s IVDs *ex vivo* model. **(A)** H&E and Safranin O-Fast Green staining of paraffin sections of the rat’s intervertebral discs *ex vivo* model at a coronal position. **(B)** Immunofluorescence analysis for phosphorylation and translocation of P65 in NP cells pretreated with 50 μM Curcumenol and stimulated with TNFα (10 ng/ml) for 20 min. **(C)** Immunohistochemistry analysis of TNFα and IL-1β expression in *ex vivo* model at a coronal position. **(D)** Quantification of histological score in the sections described in panel A. **(E)** Quantification of positive cells in the sections described in panel **(C)** *p < 0.05, **p < 0.01, ***p < 0.001 and ****p < 0.0001.

To further investigate the anti-inflammatory effect of curcumenol in NP cell line, we stimulated the cells with TNFα (10 ng/ml, 24 h) and found that the expression of MMP3, 9, and 13 dramatically increased (Figures 2B–D), with a decreased chondrogenic marker, Col2a1 (Figure 2A). If we treat the inflammatory cells with curcumenol in different concentrations ranging from 0, 6.25 to 50 μM, the expression of these MMPs and Col2a1 would be rescued, although there is no concentration-dependent effect (Figures 2A–D). Using western blot to confirm the rescue effects of curcumenol in NP cell line, we stimulated the cells with TNFα (10 ng/ml, 24 h) with or without curcumenol (50 μM). Results showed that the MMP family proteins broadly increased and curcumenol (50 μM) could effectively mitigate the up-regulation of these inflammatory proteins (Figures2F,G), in accordance with the consequence of the PCR. So we believe curcumenol is an effective compound to remodel the catabolism deteriorated by TNFα. The molecular mechanism involved might be that curcumenol inhibited the NFκB pathway activated by TNFα in NP cell line. Using western blot analysis, the phospho-P65 and phospho-IκBα significantly increased with the trigger of TNFα (10 ng/ml, 10 min) but could be inhibited by pretreatment with 50 μM curcumenol (Figures 2E,H). Moreover, curcumenol could increase the total protein of IκBα decreased by TNFα (Figure 2E). By immunofluorescence assay, with the stimulation of TNFα for 20 min, the p-p65 was activated and then translocated into the nucleus, but if we pretreated the cells with 50 μM curcumenol the translocation of p-p65 was effectively blocked (Figure 3B).

### Curcumenol Mitigated the Inflammation Induced by TNFα in the Rat’s IVD *Ex Vivo* Model

According to the research of the rat’s intervertebral discs *ex vivo* model (Haschtmann et al., 2006; Xiang et al., 2020), the intervertebral discs could keep their viability for 2 weeks in cell culture medium and acquired stimulation when added into the medium. So in our research, we used this *ex vivo* model to further confirm the anti-inflammatory effect of curcumenol. SO-FG and H&E staining showed that the discs degenerated with stimulation of TNFα, expressed as NP shrinking and disregulation of AF. But the addition of curcumenol (50 μM) could effectively rescue the phenotype of degeneration of discs based on the quantification of histological score (Figures 3A,D). Using immunohistochemistry test to explore the mechanism, we found that TNFα stimulation could up-regulate the expression of inflammatory cytokines like TNFα and IL-1β, which embarked the subsequent inflammatory reactions and led to the degeneration of IVD, but curcumenol could dramatically inhibit this effect with significance (Figures 3C,E).

### Curcumenol Mitigated Inflammatory Reactions in the Rat’s Primary Nucleus Pulposus Cells by Inhibiting the NFκB Pathway *In Vitro*

**FIGURE 4:**
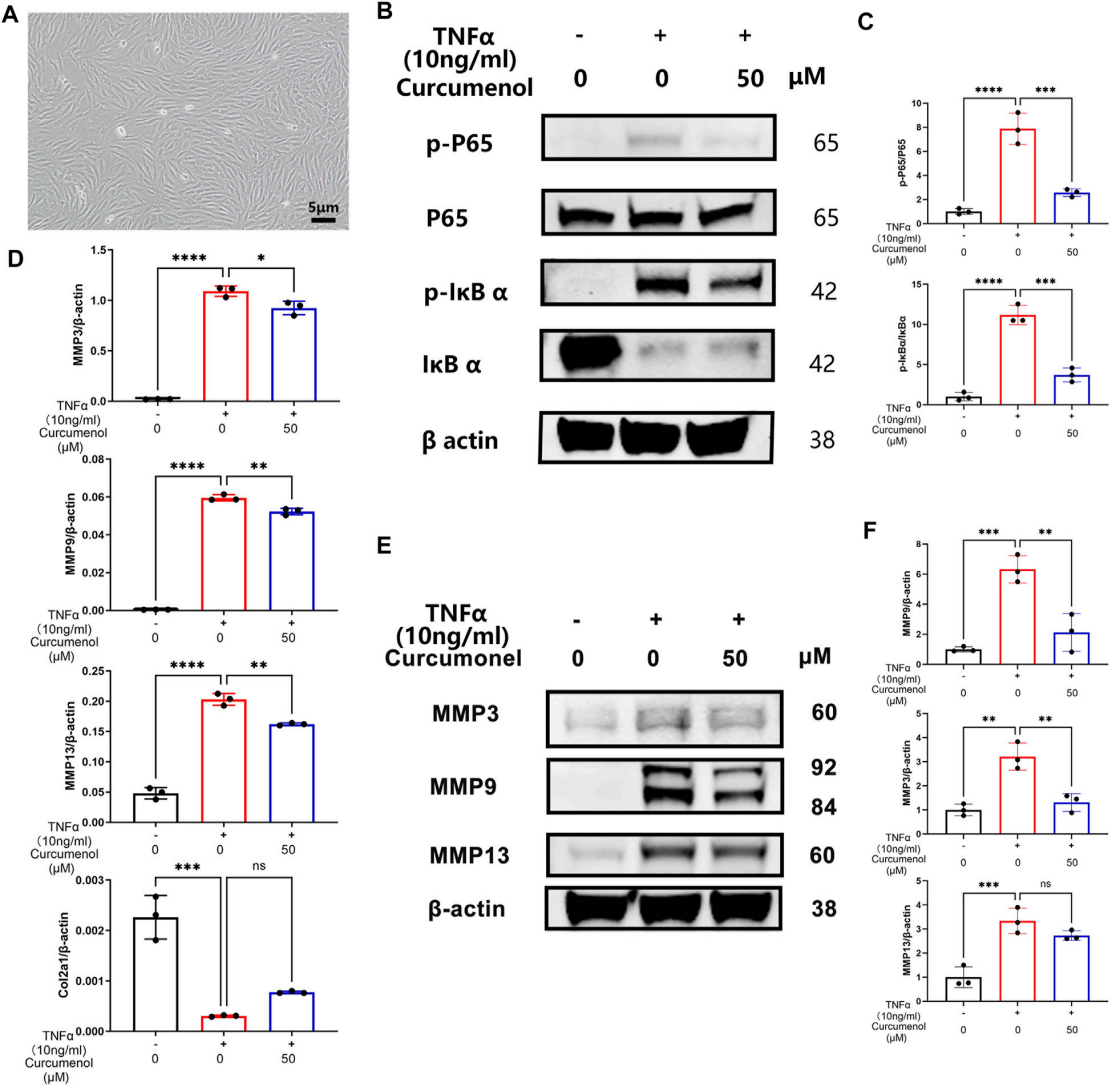
Curcumenol mitigated inflammatory reactions in rat’s primary NP cells by inhibiting the NFκB pathway *in vitro*. **(A)** Successful isolation of primary NP cells in rats. **(B)** Western blot analysis of p-P65, P65, p-IκBα, and IκBα expression in primary NP cells pretreated with 50 μM Curcumenol and then stimulated with TNFα (10 ng/ml) for 10 min. **(C)** Semi-quantification of grey scale value in p-P65/P65 and p-IκBα/IκBα was conducted in panel B. **(D)** RT-qPCR analysis of the relative mRNA expression levels of Col2a1, MMP3, MMP9, and MMP13 in primary NP cells stimulated with TNFα (10 ng/ml) and 50 μM Curcumenol for 24 h. **(E)** Western blot analysis of MMP3, MMP9, and MMP13 expression in primary NP cells stimulated with TNFα (10 ng/ml) or/and 50 μM Curcumenol for 24 h. **(F)** Semi-quantification of grey scale value in MMP3, MMP9, and MMP13 was conducted using β-actin as the reference in panel E. All data are presented as mean ± SD from three replicates. *p < 0.05, **p < 0.01, ***p < 0.001, and ****p < 0.0001. Col2a1, collagen type II α 1 chain; IOD, integrated optical density.

We tried to isolate the rat’s primary NP cells to further confirm the anti-inflammatory function of Curcumenol (Figure 4A). Curcumenol could effectively mitigate the gene up-regulation of MMP family and down-regulation of Col2a1 induced by TNFα with significance (Figure 4D), and the inner molecular mechanism was that the Curcumenol could inhibit the activation of the NFκB pathway and then finally inhibit the up-regulation of the MMP family. In our research, TNFα stimulation could activate the classical inflammation pathway NFκB, increasing the phosphorylation of P65 and IκBα immediately, while after pretreating the cells with Curcumenol this phosphorylation would be inhibited especially on the proteins p-P65 and p-IκBα with significance (Figures 4B,C). The degradation of IκBα would also be rescued at the same time (Figure 4B). Moreover, we could easily observe that the increased catabolic protein MMP family *via* TNFα stimulation were similarly down-regulated significantly (Figures 4E,F) in western blot analysis, which further confirmed our hypothesis.

### Curcumenol Rescued the Mice’s Intervertebral Disc Degeneration Induced *via* the Lumbar Spine Instability Mouse Model *In Vivo*

**FIGURE 5:**
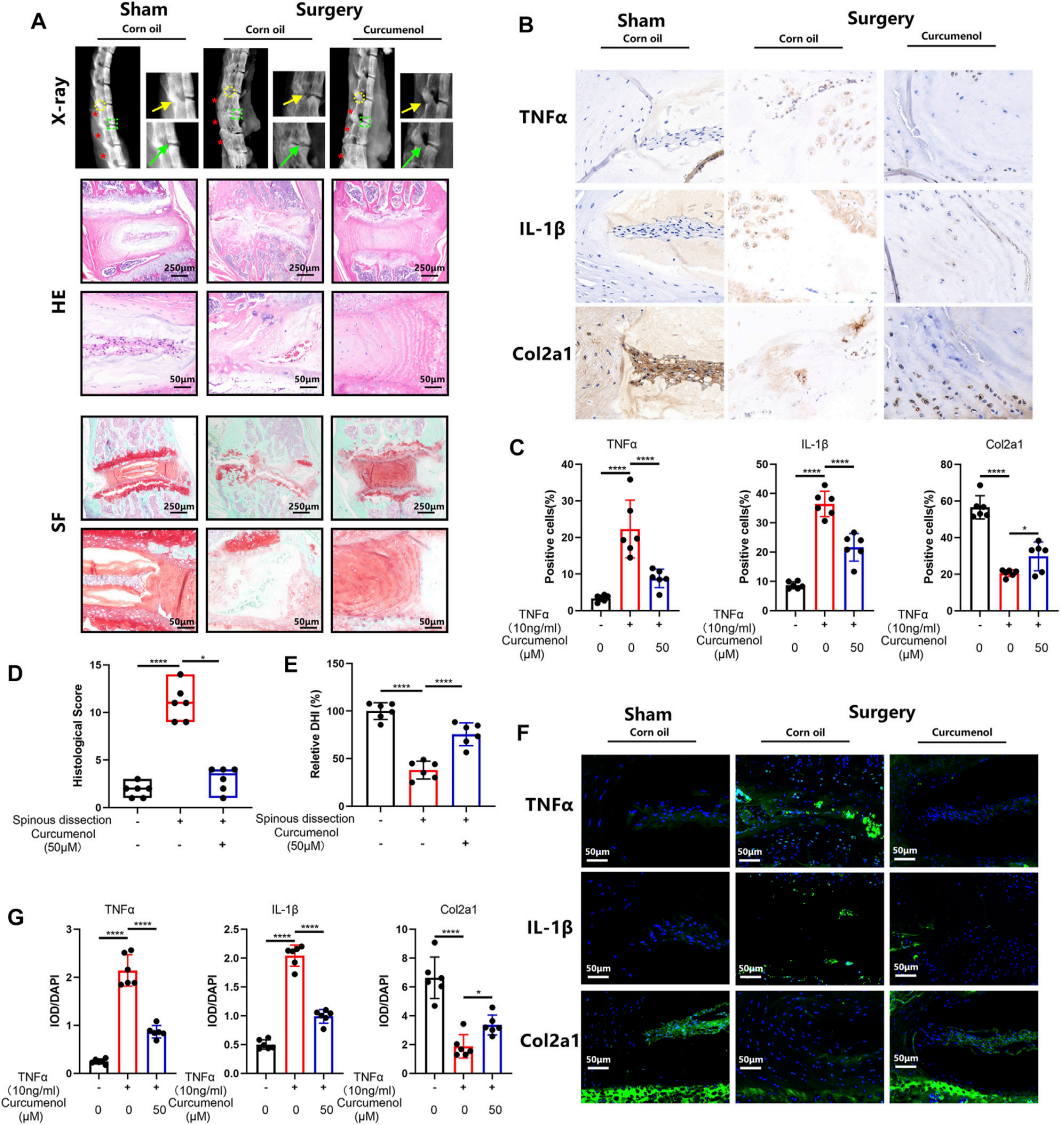
Curcumenol treated the mice’s IVDD induced *via* lumbar spine instability mouse model *in vivo*. **(A)** X-ray of lumbar spines in mice and then the images were cropped to focus on the area of operation. Safranin O-Fast Green and H&E staining of paraffin sections of the lumbar spines in mice at a coronal position. **(B)** Immunohistochemistry analysis of TNFα, IL-1β, and Col2a1 expression in lumbar spines of mice. **(C)** Quantification of positive cells in the sections described in panel B.**(D)** Quantification of histological score in the sections described in panel A. **(E)** Quantification of relative DHI in the sections described in panel A. **(F)** Immunofluorescence analysis of TNFα, IL-1β, and Col2a1 expression in lumbar spines of mice. **(G)** Quantification of IOD/DAPI in the sections described in E. *p < 0.05, **p < 0.01, ***p < 0.001, and ****p < 0.0001. Col2a1, collagen type II α 1 chain; IOD, integrated optical density. * Spinal Process; **→**Intervertebral Disc; **→**Facet Joint.

Curcumenol is not only effective just *in vitro* but also showed great rescue functions in animal models in our research. To create the degeneration model of IVD, we dissect the L2-L4 lumbar spine process of mice, making an unstable environment for lumbar motion (Ni et al., 2019). Three months after the surgery, the lumbar spines were harvested for further tests. X-ray analysis showed that the lumbar spine in the surgery group degenerated severely as compared with the sham group, expressed as osteophyte formation and loss of height in intervertebral discs, but with curcumenol administration, these discs regenerated near to normal phenotype (Figure 5A). Also, SO-FG and H&E staining showed abnormal structure of the discs after surgery, but curcumenol administration could effectively rescue this degeneration, and the quantification of histological score also showed great rescue effect of curcumenol on surgery-induced disc degeneration (Figures 5A,D). Moreover, we could observe that the quantification of DHI could also be restored with curcumenol treatment (Figure 5E). Using immunohistochemistry test to explore the mechanism, we found that the expression of inflammatory cytokines like TNFα and IL-1β increased, chondrogenic markers like Col2a1 decreased after surgery, but curcumenol administration effectively blocked these inflammatory reactions in the curcumenol group with significance (Figures 5B,C). Using an immunofluorescence assay we confirmed the same consequence that TNFα and IL-1β up-regulated with surgery, but curcumenol could effectively down-regulate the expression of TNFα and IL-1β and rescue Col2a1 significantly (Figures 5F,G).

## Discussion

Chronic LBP caused by intervertebral disc degeneration or herniation harassed more and more people in our society, which led to sub-health status, even disability, and increased at an alarming rate. Treatments of IVDD, whatever in the clinic occasion or just used in the research, including genetic therapy (Roh et al., 2021), stem cell therapy (Krut et al., 2021), somatic cell therapy (Zhang et al., 2021), and the end-stage choice, surgical intervention, were surely effective (Hu et al., 2019). However, considering that these traumatic treatments had more and more side effects like adjacent segment degeneration, cell leakage, and safety of gene therapy (Gul et al., 2020). Thus, anti-inflammatory strategy to ameliorate IVDD still needs to be further explored.

Nowadays, planet-derived traditional medicine, different from the commonly used non-steroidal anti-inflammatory drugs and some analgesics (Lo et al., 2015) with side effects like hepatic damage or gastrointestinal injury (Derry et al., 2017; Rasmussen-Barr et al., 2017), has gained much more attention in recent years for their fewer side effects, abundant production capacities, and prominent anti-inflammatory function (Chen et al., 2017; Li et al., 2020; Liu et al., 2020). Based on these theories, we got the idea to use this traditional medicine to ameliorate the symptoms and slow down the progression of IVDD (Enthoven et al., 2016). Curcumenol is a bioactive compound isolated from the edible rhizome of Curcuma zedoaria. It is used to treat synovitis of knee osteoarthritis in patients (Wang et al., 2020) showing effective anti-inflammatory function, but the exact pathways and mechanisms involved still need to be further explored. Thus, considering the broad applied range of Curcuma category, in our study we discovered the anti-inflammatory effect of Curcumenol to treat TNFα induced NP cell line and primary NP cells *in vitro*, and we got convincing results that Curcumenol could rescue the inflammation of IVD in the rat’s *ex vivo* model and mice’s lumbar spine instability model *in vivo*.

Through RNA-seq, we found the expression of several genes involved in inflammatory pathways, especially the TNF signaling pathway and Toll-like receptor signaling pathway manifested distinct changes, among which the TNF receptor associated factors (TRAFs) family, the chemokine CXC motif ligands (CXCLs) family, and IL1 receptor-like 1 (IL1RL1) draws our great attention. TRAF3 is a kind of cytoplasmic signaling adaptor protein that participates in the signal transduction of several important inflammatory pathways, such as NF-κB pathways, TNF signaling pathway, and Toll-like receptor signaling pathway, thus playing a pivotal role in regulating cell proliferation, apoptosis, and inflammatory response (Häcker et al., 2011; Zhou et al., 2021; Gissler et al., 2022; Wu et al., 2022). It was reported that the expression of CXCL10 manifested a positive correlation with the severity of IVDD due to its chemotactic function of recruiting immune cells (Jacobsen et al., 2020). However, other researchers found that CXCL10 was involved in the maintenance of AF homeostasis and plays a role in AF repair (Hegewald et al., 2012). IL1RL1, also known as ST2, is the receptor of IL33, which is a new member of the IL1 family. The up-regulation of IL33/IL1RL1 contributed to the radicular pain in patients with disc herniation and was closely related to the activation of NF-κB pathways (Huang et al., 2018). In our results, we found that after treatment with Curcumenol, the expression of TNFα-induced TRAFs and IL1RL1 were notably inhibited in mRNA and protein levels, which were consistent with its protective effects for IVD by blocking the activation of NF-κB pathway. Meanwhile, Curcumenol promoted the expression of CXCL10 and did not influence the TNFα-induced up-regulation of CXCL10. We hypothesize such a phenomenon is also related to the repair of IVD. However, due to the unclear effects of CXCL10 in IVDD, we cannot give an accurate explanation and we will focus on this point in our following research.

**FIGURE 6:**
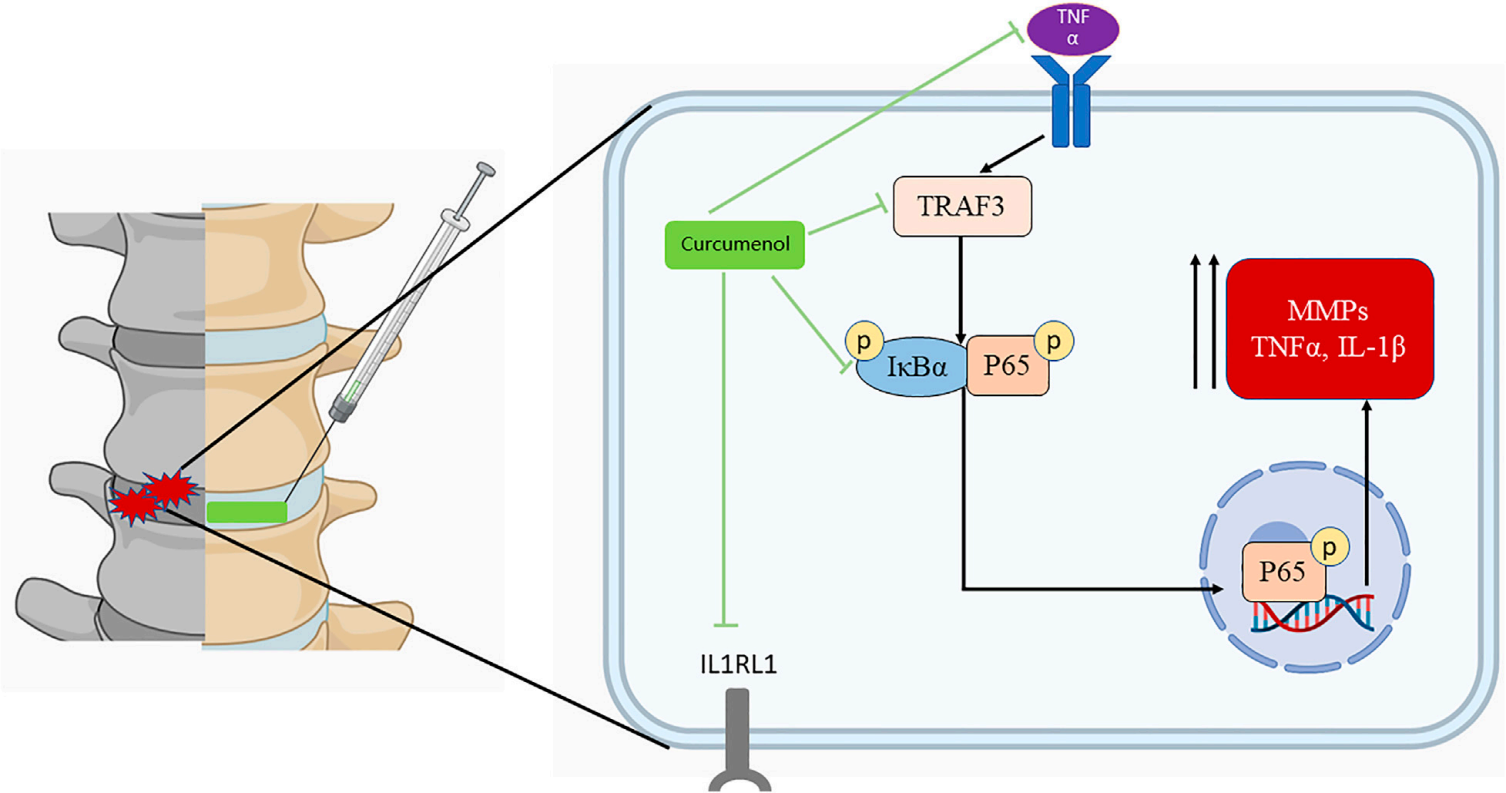
Curcumenol inhibits the inflammatory pathways, especially the TNF signaling pathway in NP cells *in vitro* and lumbar spine instability mouse model *in vivo*. In general, it inhibits the up-regulation of TRAF3 induced by TNFα, and the subsequent phosphorylation and activation of IκBα with P65. Finally, it prevents the following activation of inflammatory factors like MMP families. Created with BioRender.com

At the beginning of the degeneration in discs, inflammatory cytokines accumulate (Wu et al., 2018; Zhang et al., 2019), and one of the most important pro-inflammatory factor is Tumor Necrosis Factor α, which exerts its inflammatory effect through an important intracellular signaling pathway–NFκB pathway (Baker et al., 2011). In general, NFκB is a complex formed by seven transcription proteins and is normally located in the cytoplasm in a denatured status, bound with its inhibitor protein IκBα (Zhongyi et al., 2015). When TNFα stimuli connected to its receptor and then the inflammatory signal passed into the cells (Zhang et al., 2017), it activated the phosphorylation of an IκB kinase (IKK) complex, composed of three associated subunits, of which the IKKα and IKKβ exert the catalytic function. Phosphorylated IKKβ/α activated and phosphorylated the IκBα and then finally degraded it in a ubiquitin proteasome-dependent pathway, following this, the NFκB unbounds from the IκBα and exposes the phosphorylation locus. Phosphorylated NFκB disposed of the p65 subunit and translocated into the nucleus, where it could bind to some responsive genes and then induce the up-regulation of some inflammatory productions, catabolic enzymes, and apoptotic mediators (Baldwin, 1996; Sun et al., 2015; Tu et al., 2017; Chen et al., 2020). The MMP family plays an important role in such degeneration initiated by the NFκB pathway, as it increased and promoted the ECM degradation (Vo et al., 2013), for example, the high-level expression would degrade the type II collagen (He et al., 2020). More than that, the disturbance of anabolic and catabolic balance and the dramatically increased inflammatory cytokines may finally cause the apoptosis of NP cells, which further induced the instability and height loss in discs. Targeting on this important pro-inflammatory pathway and considering the efficient application of Curcumenol on microglial cells (Lo et al., 2015), in our study, we used Curcumenol to successfully inhibit the phosphorylation of IκBα and NFκB p65 subunit. At the same time the nucleus translocation of p-p65 was also blocked, which led to the following decrease in the MMPs and rescue in type II collagen in NP cell line and primary NP cells (Figure 6).

Curcumenol is not only useful just *in vitro.* In our study, we used an IVD *ex vivo* model to further confirm its efficiency in mimicking an *in vivo* condition. Based on previous research, the isolated IVD fragments could be cultured for at least 6 days with preserved cell viability (Dittmar et al., 2014; Xu et al., 2020), which was used for biomechanical or drug transferring-system studies (Hartman et al., 2012; Cherif et al., 2020). Curcumenol could be absorbed by the NP cells and then attenuate the destructive effects of TNFα, which were manifested by rehydrated NP tissue in an *ex vivo* model. Lumbar spine instability mouse model in mice was an effective animal model which came up recently (Zheng et al., 2018), through resecting the spinous process from the first to the fourth lumbar spine, which could harmfully affect the stable status in normal spine, leading to the heavier burden-load to the IVD located in the former spine mechanically, which could restrict the range of motion of these segments (Ni et al., 2019). Using this widely recognized mice model, we showed the rescue effect of Curcumenol in IVDD. It could effectively restore the disc height (Figure 5E), prevent the disc degeneration (Figure 5D), and even fusion under unstable and inflammatory conditions.

This study also has three limitations. First, the results of RNA-seq were not in-depth explored. Through the RNA-seq, we found that the expressions of TRAFs, CXCLs, and IL1RL1 changed obviously after the treatment of curcumenol. Although we further detected the expression of these molecules in mRNA and protein levels in our following studies and elucidated the potential relationships of these molecules with curcumenol in the discussion section, the in-depth molecular mechanism of curcumenol to treat IVDD still needs further investigation based on the results of RNA-seq. Second, the rats used in the *ex vivo* models were a little young. It is reported that the IVD will undergo a degenerative process very early after skeletal maturity (Urban and Roberts, 2003; Frapin et al., 2019). Therefore, the 3-month-old rats are suitable for the exploration of early physiological IVDD. However, the IVDD-related low back pain generally occurred in middle-aged and elderly persons, whose IVDD was more severe. To mimic such a situation, the ex vivo models using elderly rats may be more appropriate. Third, although the rat’s *ex vivo* model could confirm the treatment effects *in vivo* partially and we also verified the effects in a lumbar spine instability mouse model, we still lack a rat’s model to further illustrate the treatment effects of curcumenol *in vivo* and we will improve that in our following studies.

## Conclusion

Overall, in our study, we offered a newly extracted, plant-derived bioactive medicine, curcumenol, and we demonstrated that it may serve as a potential anti-inflammatory agent for the management of IVDD. The less cytotoxicity and side effects and high production are the main advantages if used in clinical situation in the future.

## Data Availability

The datasets presented in this study can be found in online repositories. The names of the repository/repositories and accession number(s) can be found below: ncbi.nlm.nih.gov; PRJNA845918.

## Supplementary Material

Supplementary Figure S1
**(A)** RT-qPCR analysis of the relative mRNA expression levels of CXCL1, CXCL6 and CXCL16 expression in NP cell stimulated with TNFα (10 ng/ml) or/and 50 μM Curcumenol for 24 h. **(B)** RT-qPCR analysis of the relative mRNA expression levels of TRAF1, TRAF2, TRAF4, TRAF6, and NOS2 expression in NP cell stimulated with TNFα (10 ng/ml) or/and 50 μM Curcumenol for 24 h. **(D)** RT-qPCR analysis of the relative mRNA expression levels of IL1RL1 expression in NP cell stimulated with 50 μM Curcumenol for 24 h. All data are presented as mean ± SD from three experiments. ^*^ *p* <0.05, ^**^ *p* <0.01, ^***^ *p* <0.001 and ^****^ *p* <0.0001.

## Abbreviations

AF, annulus fibrosus; CCK-8, Cell Counting Kit-8; CEP, cartilage endplate; DMSO, Dimethyl Sulfoxide; DMEM, Dulbecco’s modified Eagle’s medium; ECM, extracellular matrix; FBS, fetal bovine serum; HE, hematoxylin and eosin; IL-1β, interleukin-1β; IVDD, intervertebral disc degeneration; IVD, intervertebral disc; LBP, low back pain; MMP, matrix metalloproteinase; NP, nucleus pulposus; NF-κB, nuclear factor kappa-B; OD, optical density; PFA, paraformaldehyde; RT-qPCR, real-time quantitative PCR; RT, room temperature; SDS-PAGE, sodium dodecyl sulfatepolyacrylamide gel electrophoresis; SO-FG, safranin O-fast green; TNFα, tumor necrosis factor alpha; WB, western blotting.
